# An Evaluation of the Implementation of the European Calcified Tissue Society Recommendations on the Prevention and Treatment of Osteoporosis Secondary to Bariatric Surgery

**DOI:** 10.3390/nu15041007

**Published:** 2023-02-17

**Authors:** Marion Courtalin, Hélène Verkindt, Naima Oukhouya Daoud, Nassima Ramdane, Bernard Cortet, François Pattou, Julien Paccou

**Affiliations:** 1 Department of Rheumatology, University of Lille, 59000 Lille, France; 2 Department of Endocrine and Metabolic Surgery, University of Lille, 59000 Lille, France; 3 METRICS: Evaluation des Technologies de Santé et des Pratiques Médicales, University of Lille, 59000 Lille, France

**Keywords:** bariatric surgery, osteoporosis, recommendations, obesity, fracture, European Calcified Tissue Society, metabolic surgery

## Abstract

The purpose of this study was to evaluate the implementation of the European Calcified Tissue Society (ECTS) 2022 recommendations on the prevention and treatment of osteoporosis secondary to bariatric surgery. The ECTS 2022 recommendations were applied in a retrospective cohort of postmenopausal women and men aged 50 years and older who were undergoing or had already undergone bariatric surgery. Osteoporosis medication was indicated if any of the following criteria were met: (i) history of recent (within 2 years) fragility fracture after the age of 40 years, (ii) BMD T score ≤ −2 at any of the sites of measurement, and (iii) FRAX^®^ ≥ 20% for major osteoporotic fractures and/or ≥3% for hip fractures. Of the 170 patients (144 women, mean age 59 (55 to 63) years) included between February 2019 and March 2022, 33 were eligible for osteoporosis medication based on the ECTS 2022 recommendations, i.e., a prevalence of 19.6% [CI95%: 13.9%; 26.5%]. Most patients met the BMD T score ≤ −2 criterion (*n* = 25/170, 14.7% [CI95%: 9.7%; 20.9%]) and/or the history of recent fragility fracture criterion (*n* = 12/170, 7.1% [CI95%: 3.7%; 12.0%]). In this study, a fifth of our population was found to be eligible for osteoporosis medication after the application of the ECTS 2022 recommendations.

## 1. Introduction

The prevalence of obesity continues to increase worldwide and is an international public health concern due to the multiple co-morbidities associated with it [1]. Indeed, obesity is a major risk factor for cardiovascular diseases, diabetes, sarcopenia, falls and fractures at certain sites, and several malignant diseases [2]. According to the WHO estimates, more than 1.9 billion adults were overweight in 2016. Among them, 650 million were obese [3]. In France, the prevalence of obesity is between 10% and 20% of the population, albeit with regional disparities: the northern and eastern regions are the most affected [4,5].

In people with obesity (PwO), nutritional support and conventional medical therapies frequently reach their limits. Advances in surgical procedures to treat PwO have led to sustained weight loss and improvements in or even the elimination of some obesity-related comorbidities, such as hypertension or type 2 diabetes (T2DM) [6,7,8]. Consensual indications for bariatric surgery are body mass index (BMI) ≥ 40 kg/m^2^, BMI ≥ 35 kg/m^2^ with T2DM, or other comorbidities that could be significantly improved after bariatric surgery. Their growing popularity has led to a near-constant increase in bariatric surgery procedures worldwide [9]. In 2016, 59,300 procedures were performed in France, breaking down as follows: sleeve gastrectomy (SG, 58.5%), Roux-en-Y gastric bypass (RYGB, ∼25%), gastric-band gastroplasty (GB, <5%), and biliopancreatic diversion (BPD < 100 procedures a year). Currently, in France more than 600,000 individuals, i.e., ∼1% of the adult population, have already undergone bariatric surgery [10].

However, adverse consequences for bone health have recently been highlighted [11,12,13,14,15,16,17]. Accordingly, bariatric procedures have been found to be associated with rapid bone loss and dramatic increase in bone turnover markers (BTMs), leading to both an early and sustained bone loss [11,12]. Furthermore, this state is associated with reduced bone strength, microarchitecture deterioration, and an increased risk of fracture over time, especially with the Roux-en-Y gastric bypass (RYGB) procedure [13,14,15]. Paccou J. et al. reported a significant 1.22-fold [1.08–1.39] increase in the major fragility fracture risk in the surgical group (consisting of humerus, wrist, hip, and vertebral fractures) compared with an obese control group (matched for age, sex, grade of obesity, year of inclusion, and Charlson comorbidity index) [16]. They observed an increase in the risk of fragility fracture for RYGB only (HR = 1.70, 1.46 to 1.98) but not SG or other procedures compared with the matched controls. In the Swedish Obese Subjects (SOS) study, an ongoing, non-randomized, prospective, controlled intervention study, the authors investigated the fracture risk for different bariatric surgery procedures in 2007 patients treated with bariatric surgery and 2040 control patients [17]. The median follow-up time was 17.6 years and the highest incidence rate for first-time fracture was observed in the RYGB group (22.9 per 1000 person years). The risk of fracture was increased in the RYGB group compared with the control group (adjusted HR = 2.58, 2.02–3.31).

Furthermore, data on the evolution of bariatric surgery in France show that the proportion of PwO over 55 years of age who underwent bariatric surgery between 1997 and 2016 increased considerably, from 9.3% to 16.2% [10]. As it is known that those most at risk of developing osteoporosis are postmenopausal women and men over 50 years of age, screening for osteoporosis in this population is advisable [18,19].

Recently, in 2022, the European Calcified Tissue Society (ECTS) proposed screening for an indication of anti-osteoporosis medication (AOM) in postmenopausal women and men over 50 years of age before and after bariatric surgery, if any of the following criteria are met: (i) history of recent (within 2 years) fragility fracture after 40 years of age, (ii) BMD T score ≤ −2 at the hip and/or spine, and (iii) FRAX^®^ score ≥ 20% for 10-year major osteoporotic fractures and/or ≥3% for hip fractures [20].

We conducted this retrospective cohort study with the hypothesis that the implementation of the ECTS guidelines 2022 would be feasible and useful to screen PwO who are eligible for AOM in the context of bariatric surgery. The purpose of the study was to assess the implementation of the 2022 ECTS guidelines in a cohort of postmenopausal women and men aged 50 years and older that have already been assessed between 2019 and 2022.

## 2. Patients and Methods

### 2.1. Study Design

The2022 ECTS guidelines were applied in a retrospective cohort of postmenopausal women and men aged 50 years and older. All of the patients were PwO who had been referred by the Metabolic Surgery Department to the Rheumatology Department for an assessment of bone mineral density (BMD) using dual-energy X-ray absorptiometry (DXA) between February 2019 and March 2022.

### 2.2. Study Approval

The study protocol was approved by the Commission Nationale de l’Informatique et des Libertés (CNIL) (n°DEC2021-130), and the study procedures complied with the ethical standards of the relevant institutional and national Human Experimentation Ethics Committees. Patients’ written consent was not required due to the retrospective design of the study.

### 2.3. Study Population

Inclusion criteria: Postmenopausal women and men aged 50 years and older, followed at the Lille University Hospital. PwO encountered at the time of (1) a preoperative assessment performed prior to bariatric surgery, including (1a) patients who had no previous bariatric surgery, or (1b) patients who had already undergone at least one bariatric surgery procedure, and (2) a postoperative assessment performed after a previous bariatric surgery procedure. Patients who gave their consent to be screened for DXA assessment after clear, fair, and intelligible information. Patients who did not object to the use of their anonymized clinical data for research purposes.

Exclusion criteria: Patients who had not undergone DXA at the Lille University Hospital’s Rheumatology Department, or who presented with one or more pathologies such that it was technically difficult for a correct evaluation of BMD (e.g., severe dysplasia, hip or lumbar spine surgery, etc.). Men under 50 years of age and premenopausal women; patients weighing more than 160 kg (the accepted threshold for the bone densitometry table), or unable to move on the examination table on their own. Patients seen in the Metabolic Surgery Department for a short period of time for a specialist opinion (e.g., proposal to re-operate following a complication, feasibility opinion), and whose preoperative evaluation and postoperative follow-up took place in another hospital, were excluded from the study, as were patients whose preoperative assessment was conducted by teleconsultation only, due to the COVID-19 epidemic.

Prior use of AOM was allowed as well as current use. Moreover, diagnosis of osteoporosis was not an exclusion criterion.

### 2.4. Study Protocol

During the evaluation conducted at the Rheumatology Department, patients were systematically invited to undergo a DXA scan to assess their lumbar spine and hip BMD. During this examination, several types of data were collected by a radiology technician using a standardized questionnaire. The collected data included data on medication, history of fragility fracture, and osteoporosis risk factors.

The DXA results were then interpreted by a physician from the Rheumatology Department, who contacted patients by phone when data, especially fracture history data, were missing or incomplete.

The DXA data, the data from the standardized questionnaire collected by the radiology technician, and clinical data collected from Lille University Hospital’s computerized files, were then collected retrospectively.

Data from the ABOS (Atlas Biologique de l’Obésité Sévère) or OBESE CLASSIQUE cohort were used to complete the collection of certain patients’ clinical, anthropometric, and biological characteristics.

The ABOS study dataset includes more than 1500 PwO, exhibiting various levels of glucose tolerance and having undergone different types of bariatric surgery [21]. All of these PwO had consented to complete phenotyping prior to bariatric surgery and during their follow-up over a period of 5 years. The cohort was approved by the Lille University Hospital’s Ethics Committee (NCT01129297). Thirty-five (35) patients included in our study were from the ABOS cohort.

The OBESE CLASSIQUE cohort is a database compiled from the files of PwO operated on in the Lille University Hospital’s Metabolic Surgery Department [22]. Fifty-four (54) patients included in our study were from the OBESE CLASSIQUE cohort. The OBESE CLASSIQUE cohort was approved by the Lille University Hospital’s Ethics Committee, and the study was supported by grants from the French government and the French Ministry of Health (PHRC). Following an update to the relevant law, new approval was obtained in 2006 (no. CP06/49, NCT01129297).

#### 2.4.1. Clinical Data/Interview Data

The following information was collected: gender, age, weight, height, body mass index (BMI), patient’s medical and surgical history, usual treatment(s), Charlson’s comorbidity Index (CCI), and comorbidities, such as obstructive sleep apnea (OSA), osteoporosis, hypertension. And T2DM.

Data on risk factors for osteoporosis were collected and included: history of low energy fracture (spontaneous or low kinetic fracture ≤ one fall from height), family history of first-degree hip fracture, early menopause for women (<45 years), active or weaned smoking, chronic active or past excessive alcohol consumption, prolonged corticosteroid therapy (at least 3 months at 7.5 mg/day prednisone equivalent), and history of rheumatoid arthritis. Data on prior use of AOM were also collected.

Data on history of previous bariatric surgery were collected and included: SG, RYGB, biliopancreatic diversion with duodenal switch (BPD-DS), adjustable gastric banding (AGA), B-clamp, calibrated vertical gastroplasty, and single anastomosis duodenal–ileal bypass with sleeve gastrectomy (SADI).

#### 2.4.2. Biological Data

We collected the following biological parameters if they were available from the Metabolic Surgery Department: C-reactive protein (CRP), calcium, 25 (OH) vitamin D, intact parathyroid hormone (iPTH), creatinine and estimation of creatinine clearance, and glycated hemoglobin (HbA1C).

#### 2.4.3. Bone Mineral Density (BMD) by DXA

Bone mineral density (BMD in g/cm^2^ of hydroxyapatite) was measured at the lumbar spine (L1–L4) and at the non-dominant hip by DXA (HOLOGIC Discovery A S/N 81360). The machine was calibrated daily, and quality assurance tests were carried out daily and weekly. WHO criteria were used to define osteoporosis and osteopenia (BMD T score < −2.5 and BMD T score between −1 and −2.5, respectively).

#### 2.4.4. History of Fragility Fracture

History of fragility fracture was systematically collected by questioning during the interview with our radiology technician. In addition, we opportunistically searched for vertebral fractures on scans performed within the last 5 years, particularly abdominal and pelvic scans, if these were available.

### 2.5. Statistical Analysis

Categorical variables are expressed as numbers (percentage). Quantitative variables are expressed as mean (standard deviation, SD) or as median (interquartile range, IQR) for non-Gaussian distributions. Normality of distributions was assessed using histograms and the Shapiro–Wilk test. Comparisons of patients’ characteristics, biochemistry, comorbidities, risk factors, and BMD between groups were performed using Student’s t test for quantitative variables (or the Mann–Whitney U test for non-Gaussian distributions) and the Chi-squared test (or Fisher’s exact test when the expected cell frequency was <5) for categorical variables. Associations between treatment eligibility and predetermined risk factors (gender, age, BMI, CCI, smoking status, history of bariatric surgery, history of malabsorptive procedure, and 25 (OH) Vitamin D) were investigated by univariate analysis using Wilcoxon two-sample test for continuous risk factors, or the Chi-square/Fisher’s exact test for categorical risk factors. Statistical testing was conducted at the two-tailed α-level of 0.05. Data were analyzed using SAS software version 9.4 (SAS Institute, Cary, NC, USA).

## 3. Results

### 3.1. Demographics and Disease Characteristics

In this cohort of 170 patients (*n* = 144 women, 84.7%), the median (IQR) age was 59 (55 to 63) years. The median BMI was 37.7 (32 to 42.1) kg/m^2^, and 75 patients (44.1%) had a BMI ≥ 40 kg/m^2^. Table 1 shows the patients’ baseline characteristics. A total of 96 patients had undergone DXA prior to bariatric surgery (Group 1, preoperative status). Of those 96 patients, 30 had already undergone at least one bariatric surgery procedure (Group 1b). A total of 74 patients had undergone DXA as part of a postoperative follow-up without a new planned procedure (Group 2, postoperative status). BMI was significantly higher in Group 1 than in Group 2 (40.2 (35.9 to 44) kg/m^2^, and 32.6 (28 to 38.7) kg/m^2^, respectively; *p* < 0.001). CCI was lower in Group 2 (*p* = 0.002). The most frequently encountered comorbidities were hypertension (2 = 109, 64.1%), OSA (*n* = 93, 54.7%), and T2DM (*n* = 60, 35.3%) (Table 1). The diagnosis of osteoporosis was not encountered. In the whole population, the main risk factors for osteoporosis were active smoking (*n* = 19, 11.2%), early menopause (*n* = 30, 29.4%), previous exposure to prolonged oral corticosteroid therapy (*n* = 20, 11.8%), and excessive alcohol consumption (*n* = 10, 5.9%). Family history of first-degree hip fractures was observed in 12 patients (7.1%). Prior or current use of AOMs was not found.

The mean value of 25 (OH) vitamin D in the whole population was low, with a median of 27 (18 to 33) ng/mL (Supplementary Table S1). No statistical differences in 25 (OH) vitamin D levels were found between the two groups (*p* = 0.057).

**Figure 1:**
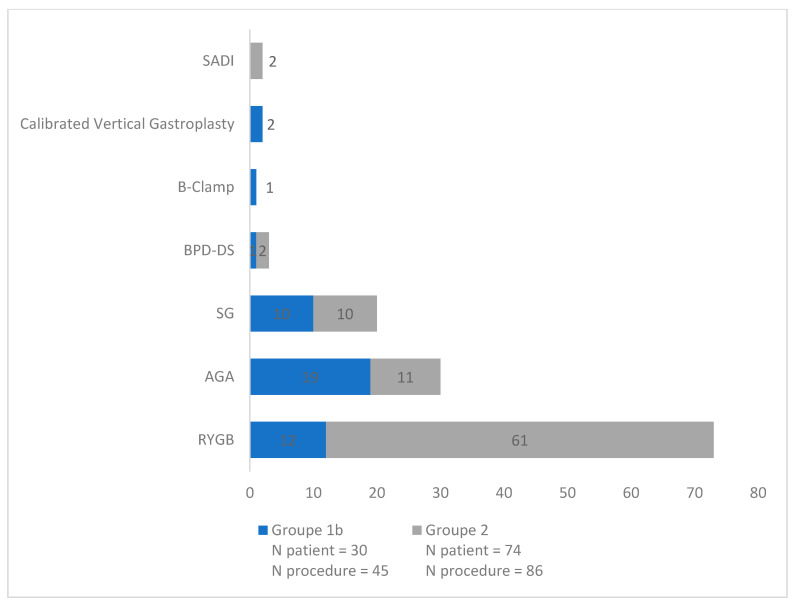
Type of surgery by frequency (*n* = 131).

A total of 131 interventions were recorded in 104 patients (Group 1b: *n* = 30; Group 2: *n* = 74). The most frequently performed procedures were RYGB (*n* = 73, 55.7%), AGA (*n* = 30, 22.9 %), and SG (*n* = 20, 15.2%) (Figure 1). When all malabsorptive surgeries (RYGB, BPD-DS, and SADI) were pooled, they accounted for 59.5% (*n* = 78/131) of the procedures performed. In patients who had undergone multiple surgeries (≥2, *n* = 27/104), the most frequent sequence was AGA followed by RYGB (*n* = 13/27, 48.1% of patients).

### 3.2. History of Fragility Fracture

History of fragility fractures after the age of 40 was self-reported by 42 patients (1/4 of our population), with a total of 49 fractures, including 16 ankle/leg fractures, 11 forearm/wrist fractures, 8 proximal humeral fractures, 2 vertebral fractures (which were confirmed by spinal imaging), 4 rib fractures, 3 pelvis fractures, 2 hip fractures, and 3 foot fractures (Table 2). History of fragility fractures was significantly less frequent in Group 1 than in Group 2 (*n* = 17/96, 17.7%, and *n* = 25/74, 33.8%, respectively; *p* = 0.016) (Table 1).

Regarding the history of recent (within 2 years) fragility fractures, 17 fractures were observed in 12 patients. The primary fracture site was the ankle/leg (*n* = 4), followed by the forearm/wrist (*n* = 3), and the proximal humerus (*n* = 3) (Table 2).

Opportunistic screening for vertebral fractures based on spinal scans was possible for 115/170 patients (67.7%). An unknown vertebral fracture was diagnosed in 3/115 patients (2.6%).

### 3.3. DXA Evaluation

In the whole population, the prevalence of osteoporosis and osteopenia for all sites was 5.4% [CI95%: 2.5%; 10.0%] and 39.4% [CI95%: 32.0%; 47.2%], respectively. Twenty-five (25) patients had a T score ≤ 2 at one of the sites, i.e., a prevalence of 14.7% [9.7%; 20.9%]. Table 3 shows the DXA results.

### 3.4. Fulfillment of ECTS 2022 Recommendations Criteria

In the whole population, 33 patients were eligible for AOMs based on the ECTS recommendations, i.e., a prevalence of 19.6% [CI95%: 13.9%; 26.5%].

When we examined the ECTS criteria in detail, most patients were found to meet the BMD T score ≤ −2 criterion at one or both sites (*n* = 25/170, 14.7% [IC95%: 9.7%; 20.9%]) (Table 4).

A small number of patients (*n* = 3/170) met the FRAX^®^ criterion (i.e., a prevalence of 2.0% [IC95%: 0.4%; 5.7%]), whereas 12/170 patients met the history of recent fragility fracture criterion (i.e., a prevalence of 7.1% [IC95%: 3.7%; 12.0%]).

The FRAX^®^ score could not be calculated in 20 patients because their weight was over 125 kg (the score is considered valid only in patients weighing less than 125 kg). Missing items were regarded as absent.

When the two groups were compared, no difference was found in the number of patients fulfilling the ECTS criteria (Group 1, 19.8%; Group 2, 19.4%; *p* = 0.96).

### 3.5. Overlap in ECTS Recommendations Criteria

**Figure 2:**
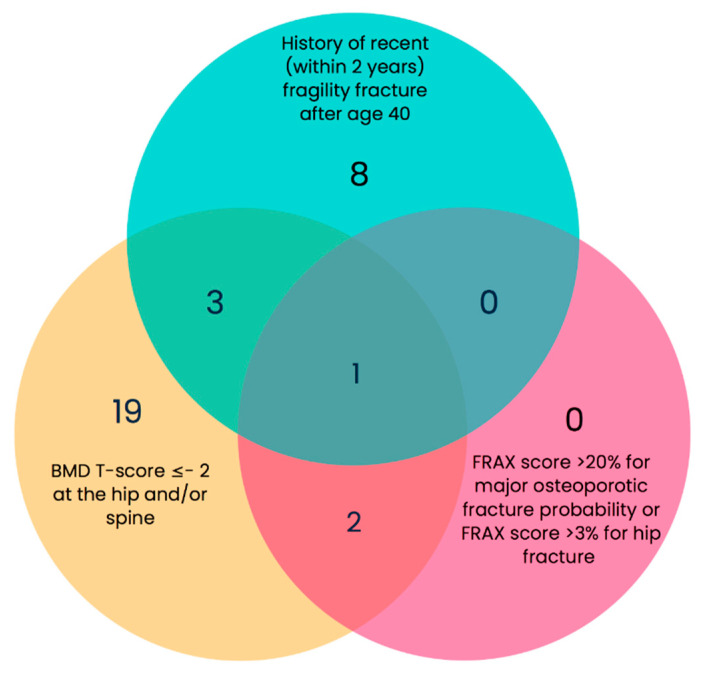
Venn diagram showing the actual number of people with obesity (PwO) for whom anti-osteoporotic medication would be recommended based on the ECTS recommendations.

Venn diagrams representing the overlap between the three ECTS criteria are presented in Figure 2. Although a substantial overlap between the different criteria might be expected in patients, the figure reveals that only one patient met all three criteria. Most patients met only a single criterion, and in most of those patients, the single criterion was a BMD T score ≤ −2 (*n* = 19). Only eight patients met the history of recent fragility fracture criterion alone. No patients met the FRAX^®^ criteria alone.

### 3.6. Relationships between Predetermined Risk Factors and Treatment Eligibility

Supplementary Table S2 shows the results of univariate analysis of predetermined risk factors and treatment eligibility. Among the risk factors, a lower BMI was the only factor associated with treatment eligibility (33.3 (29.8 to 39.1) kg/m^2^ versus 38.3 (33.1 to 42.6) kg/m^2^, *p* = 0.011).

## 4. Discussion

To the best of our knowledge, this is the first study investigating the application of the ECTS 2022 recommendations on the prevention and treatment of osteoporosis secondary to bariatric surgery. These recommendations should be applied for all patients with an indication for, or those who have already undergone bariatric surgery. Our study shows that a high percentage of patients were eligible to receive AOM. Indeed, one fifth of the whole population met the ECTS 2022 criteria. No difference was observed between patients awaiting bariatric surgery and those who had already undergone bariatric surgery.

Despite the considerable number of bariatric surgery procedures performed each year worldwide, and the negative impact of bariatric surgery on bone health [23,24], there were no recommendations on the prevention and treatment of osteoporosis secondary to bariatric surgery until recently. The ECTS published its first set of recommendations at the beginning of 2022 [20], and this study is the first to evaluate its applicability and usefulness in a targeted population of postmenopausal women and men over 50 years of age.

Only a few studies have been published on the prevalence of low BMD using a DXA assessment and the history of fragility fractures in the context of bariatric surgery in PwO [25,26]. In 2019, Blom-Høgestøl et al. sought to evaluate the prevalence of osteopenia, osteoporosis, and fragility fractures in a cohort of 124 patients 10 years after RYGB [25]. The mean (SD) age of their patients was 50.3 (9.0) years, and 94 of them (77%) were female, of which 41 (44%) were postmenopausal. Compared to our study, their patients were younger and included premenopausal women, and only one type of bariatric procedure was evaluated. In the 59 participants who were either postmenopausal females or males aged 50 years and older, the prevalence of osteopenia and osteoporosis was 51% (*n* = 30) and 27% (*n* = 16), respectively. No data on the prevalence of BMD T score ≤ −2 were available. Furthermore, the prevalence of fragility fractures was high. In postmenopausal women and men aged 50 years and older, 11 participants (19%) reported a history of fragility fracture, and the assessment of vertebral fractures by DXA revealed that 5 participants (8%) had sustained at least one moderate-to-severe morphometric vertebral fracture [25]. The findings published by Blom-Høgestøl et al. on fragility fractures are quite similar to ours. In 2014, Hintze LJ et al. published a study on 61 women (mean (SD) age: 44 (11) years) who had undergone or were awaiting bariatric surgery [26]. In the group of 40 women who had undergone bariatric surgery, the prevalence of osteoporosis and osteopenia at the lumbar spine was 10% and 17.5%, respectively. No data on the history of fragility fracture were available in that study.

Our results showed that the BMD T score ≤ −2 criterion had a major impact on treatment decisions, since many patients met the therapeutic criteria based on a BMD measurement only (19 patients (60%) eligible for treatment). This highlights the fact that this examination should be performed routinely in this target population (i.e., patients with an indication for or who have already undergone bariatric surgery). It is feasible and useful in the context of bariatric surgery, both for PwO and clinicians. Interestingly, only one patient met all three ECTS criteria for the recommendation of AOM. We found that the prevalence of patients for whom treatment would be recommended based on the FRAX^®^ criterion was low (2%). However, the FRAX^®^ score was not calculated in 20 patients, as their weight was greater than 125 kg. The fact that the FRAX^®^ score was not calculated for some patients may have underestimated the number of patients eligible for treatment. The history of recent fragility fractures (vertebral and nonvertebral) after 40 years of age is another major criterion since, for eight patients (i.e., 24% of patients eligible for treatment), the therapeutic decision was based on this criterion alone.

A few international recommendations on the management and treatment of osteoporosis secondary to bariatric surgery have recently been published [27,28,29]. The American Society for Metabolic and Bariatric Surgery (ASMBS) updated its recommendations in 2021 [27,28]. Assessing the fracture risk using bone density measurements is recommended in four cases: (1) in women aged 65 years and older, and in men aged 70 years and older; (2) in menopausal women and in men aged 50 to 69 years, depending on their fracture risk; (3) in men over 50 years of age with a history of fragility fractures as adults; and (4) in RYGB and BPD patients, regardless of age. The ASMBS recommendations do not mention which patients should be treated and how. The *Groupe de Recherche et d’Information sur les Ostéoporoses* (Osteoporosis Research and Information Group, GRIO) and the *Société Française de Rhumatologie* (French Rheumatology Society, SFR) published their first set of recommendations at the end of 2022 [29]. In the GRIO–SFR recommendations, assessing the fracture risk using bone density measurements is not recommended in menopausal women and men aged 50 years and older. An initial assessment of the fracture risk should also be routinely performed, ideally before the first bariatric surgery procedure, (i) in RYGB and BPD patients, regardless of age, and (ii) in patients at high risk of fracture, regardless of age [29]. In the GRIO–SFR recommendations, AOMs are indicated for menopausal women and men aged 50 years and older who have (i) a history of a severe fracture, regardless of T score, (ii) a history of a non-severe fracture and a T score ≤ −1, and (iii) no history of fractures and a T score ≤ −2. However, due a lack of data, they recommend against using FRAX^®^ when the weight exceeds 125 kg and consider that FRAX^®^ should be used only when it is difficult to reach a decision [29]. Interestingly, in our study we found no additional value in using FRAX^®^ after screening based on the T score ≤ −2 and/or history of recent (within 2 years) fragility fracture.

### Strengths and Limitations

The significant strengths of our study include the fact that (i) all BMD measurements were performed on the same machine by a single radiology technician, and were interpreted by a single rheumatologist, both of whom are experienced in managing PwO, and that (ii) all medical records were systematically reviewed by a single investigator. The missing data were infrequent, except for biological data.

We acknowledge several limitations to our study, including its retrospective nature. However, patients were included prospectively, with the analyses performed retrospectively. We also acknowledge that self-reported fracture history may have led to misclassification. Moreover, pooling patients awaiting bariatric surgery and those who had already undergone surgery might be seen as a limitation. However, the ECTS 2022 recommendations are applicable in both of these groups of PwO. We acknowledge that the absence of a control group precludes any definitive conclusions on the high percentage of patients eligible to receive AOM in the context of bariatric surgery.

Finally, the generalizability of our results is limited by its single-center design. However, the participants in this study represent a well-characterized population cohort of PwO, who are most often followed up in specialist obese centers such as the Lille University Hospital.

## 5. Conclusions

We found one fifth of the whole population of postmenopausal women and men aged 50 years and older for whom AOM would be recommended based on the ECTS 2022 guidelines in the context of bariatric surgery. We believe that clinicians should focus their attention on patients at a high risk of fractures. DXA should be used to measure BMD before and/or after bariatric surgery, and risk factors for osteoporosis such as history of fragility fracture should be assessed. An assessment of the efficacy of AOMs in PwO in the context of obesity is warranted.

## Figures and Tables

**Table 1:** Patients’ general characteristics at baseline.

	N	Total	N	Group 1	N	Group 2	*p*-Value
		(*n* = 170)		(*n* = 96)		(*n* = 74)	
Age (years)	170	59 (55 to 63)	96	59 (54.5 to 62)	74	60 (55 to 65)	0.17
Women (N)	170	144 (84.7)	96	81 (84.4)	74	63 (85.1)	0.89
Weight (kg)	170	99.9 (86.4 to 115)	96	107 (95 to 117.5)	74	88 (79 to 102)	<0.001
Height (cm)	170	164 (158 to 169)	96	164 (158 to 168.5)	74	163.5 (159 to 170)	0.77
Body mass index (kg/m^2^)	170	37.7 (32 to 42.1)	96	40.2 (35.9 to 44)	74	32.6 (28 to 38.7)	<0.001
Comorbidities							
Hypertension	170	109 (64.1)	96	63 (65.6)	74	46 (62.2)	0.64
Obstructive Sleep Apnea	170	93 (54.7)	96	58 (60.4)	74	35 (47.3)	0.088
Type 2 Diabetes	170	60 (35.3)	96	40 (41.7)	74	20 (27.0)	0.048
Charlson Comorbidity Index	133	2 (1 to 3)	88	2 (1 to 3)	45	1 (0 to 2)	0.002
Osteoporosis risk factors							
Excessive alcohol consumption	170	10 (5.9)	96	5 (5.2)	74	5 (6.8)	0.75
Current smoking	170	19 (11.2)	96	13 (13.5)	74	6 (8.1)	0.26
History of low energy fracture	170	42 (24.7)	96	17 (17.7)	74	25 (33.8)	0.016
Family history of hip fracture	170	12 (7.1)	96	11 (11.5)	74	1 (1.4)	0.011
Previous use of corticosteroids	170	20 (11.8)	96	13 (13.5)	74	7 (9.5)	0.41
Early menopause	102	30 (29.4)	61	17 (27.9)	41	13 (31.7)	0.68
Malabsorptive disorders *	170	59 (34.7)	96	17 (17.7)	74	42 (56.8)	<0.001
Chronic inflammatory rheumatism	170	11 (6.5)	96	8 (8.3)	74	3 (4.1)	0.35

Values expressed as number (%) or median (IQR). Abbreviations: IQR = interquartile range; * gastrectomy, extensive intestinal resections, inflammatory bowel diseases, malabsorption syndromes, and celiac disease.

**Table 2:** Patients’ fragility fracture characteristics at baseline.

	History of Low Energy Fracture > 2 Years			History of Recent Low Energy Fracture ≤ 2 Years		
	Total	Group 1	Group 2	Total	Group 1	Group 2
	N = 30	N = 13	N = 17	N = 12	N = 4	N = 8
Fragility fractures, N	32	14	18	17	4	13
Ankle/leg fractures, n (%)	12 (37.5)	9 (64.3)	3 (16.7)	4 (23.5)	0	4 (30.8)
Forearm/wrist fractures, n (%)	8 (25)	2 (14.3)	6 (33.3)	3 (17.6)	2 (50)	1 (7.7)
Proximal humerus fractures, n (%)	5 (15.6)	0	5 (27.8)	3 (17.6)	0	3 (23.1)
Vertebral fractures, n (%)	1 (3.1)	1 (7.1)	0	1 (5.9)	0	1 (7.7)
Rib fractures, n (%)	3 (9.4)	2 (14.3)	1 (5.6)	1 (5.9)	0	1 (7.7)
Pelvis fractures, n (%)	1 (3.1)	0	1 (5.6)	2 (11.8)	1 (25)	1 (7.7)
Hip fractures, n (%)	1 (3.1)	0	1 (5.6)	1 (5.9)	0	1 (7.7)
Foot fractures, n (%)	1 (3.1)	0	1 (5.6)	2 (11.8)	1 (25)	1 (7.7)

**Table 3:** Prevalence of osteoporosis, osteopenia, and T score ≤ −2.

		N	Total	N	Group 1	N	Group 2	*p*-Value
			(*n* = 170)		(*n* = 96)		(*n* = 74)	
All sites	Osteoporosis	170	5.4 [2.5; 10.0]	96	6.4 [2.4; 13.4]	74	4.1 [0.9; 11.5]	0.73
	Osteopenia		39.4 [32.0; 47.2]		38.5 [28.8; 49.0]		40.5 [29.3; 52.6]	0.79
	T score ≤ −2		14.7 [9.7; 20.9]		18.8 [11.5; 28.0]		9.5 [3.9; 18.5]	0.09
Total hip	Osteoporosis	167 ^1^	2.4 [0.7; 6.0]	96	2.1 [0.3; 7.3]	71	2.8 [0.3; 9.8]	-
	Osteopenia		16.2 [10.9; 22.6]		13.5 [7.4; 22.0]		19.7 [11.2; 30.9]	0.28
	T score ≤ −2		3.6 [1.3; 7.6]		4.2 [1.2; 10.3]		2.8 [0.3; 9.8]	-
Femoral neck	Osteoporosis	167 ^1^	3.0 [1.0; 6.9]	96	3.1 [0.7; 8.9]	71	2.8 [0.3; 9.8]	-
	Osteopenia		31.7 [24.8; 39.4]		29.2 [20.3; 39.3]		35.2 [24.2; 47.5]	0.41
	T score ≤ −2		7.8 [4.2; 12.9]		8.3 [3.7; 15.8]		7.0 [2.3; 15.7]	0.76
Lumbar spine	Osteoporosis	169 ^2^	3.6 [1.31; 7.6]	95	3.2 [0.7; 8.9]	74	4.0 [0.8; 11.4]	-
	Osteopenia		21.9 [15.9; 28.9]		27.4 [18.7; 37.5]		14.9 [7.7; 25.0]	0.05
	**T score ≤ −2**		7.1 [3.7; 12.1]		8.4 [3.7; 15.9]		5.4 [1.5; 13.3]	0.45

^1^ BMD lumbar spine measurements were not performed in 1 patient (spine surgery). ^2^ BMD hip measurements were not performed in 3 patients.

**Table 4:** Prevalence of postmenopausal women and men aged 50 years and older meeting criteria for anti-osteoporosis medication based on the 2022 European Calcified Tissue Society (ECTS) Recommendations.

	N	Total (*n* = 170)	N	Group 1 (*n* = 96)	N	Group 2 (*n* = 74)	*p*-Value
History of recent low energy fracture	170	7.1 [3.7; 12.0]	96	4.2 [1.2; 10.3]	74	10.8 [4.8; 20.2]	0.09
BMD T score ≤ −2 at one or both sites	170	14.7 [9.7; 20.9]	96	18.8 [11.5; 28.0]	74	9.5 [3.9; 18.5]	0.09
FRAX^®^ score ≥ 3% for hip fracture and/or FRAX^®^ score ≥ 20% for the 10-year major osteoporotic fracture probability	150 ^1^	2.0 [0.4; 5.7]	80	1.3 [0.03; 6.8]	70	2.9 [0.04; 9.9]	-
Eligible for osteoporosis treatment	170	19.6 [13.9; 26.5]	96	19.8 [12.4; 29.2]	72	19.4 [11.1; 30.5]	0.96

^1^ The Frax^®^ score was not calculable in 20 patients as their weight was >125 kg.

## Data Availability

The data that support the findings of this study are available on request from the corresponding author, Paccou J.

## Supplementary Materials

The following supporting information can be downloaded at: https://www.mdpi.com/article/10.3390/nu15041007/s1. Table S1: Biological data; Table S2: Relationships between predetermined risk factors and treatment eligibility.
