# Supplementary material for: An Evaluation of the Implementation of the European Calcified Tissue Society Recommendations on the Prevention and Treatment of Osteoporosis Secondary to Bariatric Surgery
Source: Nutrients. 2023 Feb 17;15(4):1007. doi: 10.3390/nu15041007 (PMC9964124; doi:10.3390/nu15041007)
Supplement: Supplementary file 1 [file nutrients-15-01007-s001.zip › nutrients-2208317-supplementary.pdf]

Supplementary Table S1: Biological data

	N	Total (n=170)	N	Group 1 (n=96)	N	Group 2 (n=74)	p-value
**BIOCHEMISTRY RESULTS**							
**Calcium [mmol/l]**	123[1]	94 (91 to 97)	79	94 (91 to 97)	44	94 (89.5 to 96.5)	0.38
**25(OH) vitamin D [ng/ml]**	145[2]	27 (18 to 33)	88	26 (17 to 31.5)	57	28 (22 to 34)	0.057
**Serum PTH [pg/ml]**	117[3]	52 (39 to 74)	74	54.5 (40 to 80)	43	45 (36 to 64)	0.17
**Creatinine [µmol/l]**	105[4]	7 (6 to 9)	74	7.5 (7 to 9)	31	7 (6 to 9)	0.076
**Hs-CRP [mg/l]**	106[6]	3.6 (1.4 to 6.0)	72	3.8 (1.4 to 7.4)	34	3.1 (1.4 to 6)	0.71
**Hba1c [%]**	134[7]	5.8 (5.4 to 6.5)	85	5.8 (5.5 to 6.7)	49	5.5 (5.3 to 6.1)	**0.049**

Values expressed as number (%). mean ± SD or median (IQR); Abbreviations: SD = Standard Deviation; IQR = Interquartile Range; PTH = parathyroid hormone

[1] Calcium measurements were not available in 47 patients

[2] 25(OH) vitamin D measurements were not available in 25 patients

[3] Serum PTH measurements were not available in 53 patients

[4] Creatinine measurements were not performed in 65 patients

[6] Hs-CRP measurements were not available in 64 patients

[7] Hba1c measurements were not performed in 36 women.

Supplementary Table S2: Relationships between predetermined risk factors and treatment eligibility

	ECTS Guidelines		
**Subgroups**	**Not eligible for osteoporosis treatment (% or Mean ± SD) N=137**	**Eligible for osteoporosis treatment (% or Mean ± SD) N=33**	**P-value**
**Gender**			

Men	21 (15.6)	4 (12.1)	0.79
Women	114 (84.4)	29 (87.9)	
**Smoking**			
Yes	12 (8.9)	7 (21.2)	0.063
No	123 (91.1)	26 (78.8)	
**History of bariatric surgery**			
Yes	78 (57.8)	23 (69.7)	0.21
No	57 (42.2)	10 (30.3)	
**History of malabsorptive surgery**			
Yes	55 (71.4)	18 (78.3)	0.52
No	22 (28.6)	5 (21.7)	
**Age**	58 (55 to 63)	60 (57 to 65)	0.21
**Body Mass Index**	38.3 (33.1 to 42.6)	33.3 (29.8 to 39.1)	**0.011**
**25(OH) vitamin D**	27 (18 to 32)	26 (18 to 34)	0.85
**Charlson Comorbidity Index**	2 (1 to 3)	1.5 (1 to 3)	0.67

*Values expressed as number (%) and median (IQR)

IQR = Interquartile Range
